# OLDER ADULT SUBSTANCE USE DISORDER TREATMENT LANDSCAPE 2020: CLIENT-LEVEL AND FACILITY-LEVEL FACTORS

**DOI:** 10.1093/geroni/igad104.2518

**Published:** 2023-12-21

**Authors:** Keith Morgen

**Affiliations:** Centenary University, Hackettstown, New Jersey, United States

## Abstract

Older adult substance use disorders (SUD) care requires specialized clinical practice. This poster reviews two datasets to emphasize client-level and facility-level factors (overall and state-by-state) relevant to older adult treatment programming, co-occurring disorders care, and days waiting for admission to treatment. The 2020 data from the National Survey of Substance Abuse Treatment Services (N-SSATS), administered by The Center for Behavioral Health Statistics and Quality (CBHSQ) of the Substance Abuse and Mental Health Services Administration (SAMHSA), U.S. Department of Health and Human Services, collects data from public/private SUD treatment facilities in the United States. Data from 16,066 facilities demonstrated only 24.7% of all facilities reported programming tailored for older adult care, with state-by-state analysis showing a range of 4.6% to 61.4% of treatment programs reporting older adult tailored care. The deficits of older adult programming availability are explained against the context of the 2020 Treatment Episode Dataset-Admissions (TEDS-A), administered by the CBHSQ, that examines admissions data from all 50 states, as well as the District of Columbia, U.S. territories, and Compact of Free Association (COFA) partners. The TEDS-A data consisted of 1,416,357 admissions, of which 11.8% (n=167,200) were aged 55 years-old or older. Of this older adult sample, 32.8% reported a co-occurring SUD and mental health disorder and 13.9% reported some number of days waiting to enter treatment. These (and other) client-level and facility-level factors (overall and state-by-state) will be used to emphasize specific needs for older adult SUD care.

